# The Impact of Follicular Fluid Oxidative Stress Levels on the Outcomes of Assisted Reproductive Therapy

**DOI:** 10.3390/antiox12122117

**Published:** 2023-12-14

**Authors:** Yu Chen, Jiahao Yang, Ling Zhang

**Affiliations:** Institute of Reproductive Health, Tongji Medical College, Huazhong University of Science and Technology, No. 13 Hangkong Road, Wuhan 430030, China; m202175750@hust.edu.cn (Y.C.); jiahaoy393@gmail.com (J.Y.)

**Keywords:** oxidative stress, follicular fluid, assisted reproductive technology

## Abstract

Oocyte quality is a pivotal determinant of assisted reproductive outcomes. The quality of oocytes is intricately linked to their developmental microenvironment, particularly the levels of oxidative stress within the follicular fluid. Oxidative stress levels in follicular fluid may have a substantial influence on oocyte health, thereby impacting the outcomes of ART procedures. This review meticulously explores the intricate relationship between oxidative stress in follicular fluid and ART outcomes. Furthermore, it delves into strategies aimed at ameliorating the oxidative stress status of follicular fluid, with the overarching goal of enhancing the overall efficacy of ART. This research endeavors to establish a robust foundation and provide valuable guidance for clinical treatment approaches, particularly in the context of infertile women, including those of advanced maternal age.

## 1. Introduction

In recent years, infertility rates have surged, attributed to a combination of factors, including delayed childbearing, shifts in lifestyle, and escalating environmental contamination [1]. Research indicates that infertility is poised to affect roughly 15% of the global population [2], underscoring it as a substantial worldwide health concern.

The principal approach to combating infertility lies in assisted reproductive technology, with the goal of enhancing its success rate shared by medical practitioners and patients alike. Crucial to the effectiveness of assisted reproductive technology is the quality of oocytes, which thrive within the intricate microenvironment of ovarian follicles. Here, follicular fluid assumes a central role in nurturing oocyte health. Oxidative stress emerges as a pivotal factor, influencing cellular function and driving cellular senescence and apoptosis. This review meticulously scrutinizes the ramifications of oxidative stress levels within follicular fluid on the developmental competence of oocytes and the outcomes of assisted reproductive therapy. Furthermore, it provides a comprehensive summary of existing strategies aimed at ameliorating the oxidative stress milieu within follicular fluid. When searching for keywords in the NCBI database, more than 50,000 articles on reproduction and oxidative stress from 1944 to the present are found, of which about 30,000 articles are on the relationship between female reproduction and oxidative stress, including 1075 clinical trials and 768 randomized controlled trials. Of the 30,000 articles, about 376 articles related to the follicular fluid have been published, including 68 mechanistic research articles and 48 review articles. This paper summarizes 90 studies that had a significant impact on oxidative stress in follicular fluids and provides a review.

## 2. Generation of Reactive Oxygen Species in Human Follicular Fluid and Its Clinical Significance

The ovarian follicle, comprising an oocyte and surrounding granulosa cells, serves as the fundamental structural and functional unit of the ovaries. As follicles develop into antral follicles, the follicular fluid (FF) fills the follicular cavity, creating a vital microenvironment for oocyte and follicle development [3]. FF is composed of plasma exudates, granulosa cell metabolic products, plasma proteins, various hormones, and paracrine growth factors. The composition of FF dynamically changes during follicular development, influenced by the hypothalamic–pituitary–gonadal axis hormones and paracrine signals within the follicular microenvironment, and indirectly affected by various pathologies [4]. These components have been shown to impact oocyte quality, early embryo development, and pregnancy outcomes [5,6,7]. Among the numerous components in FF, those associated with oxidative stress (OS) are considered some of the most crucial factors adversely affecting the outcomes of assisted reproductive technologies (ART) [8]. Research by Liu et al. indicated that polycystic ovary syndrome (PCOS) is associated with elevated OS markers in both serum and FF. Moreover, FF OS biomarkers show a higher correlation with embryo quality than serum markers, providing more accurate predictions of embryo quality [9]. A study by Artini et al. found that FF extracted from fertilized oocytes exhibited higher antioxidant capacity compared to unfertilized oocytes, directly influencing embryo development [10].

Reactive oxygen species (ROS), generated during mitochondrial electron transport for energy production, including superoxide anion (O^2−^), hydroxyl radicals (·OH), and hydrogen peroxide (H_2_O_2_), play vital roles in regulating follicular growth, oocyte maturation and ovulation, fertilization, embryo implantation, and fetal development [11]. For instance, ROS can promote angiogenesis within the ovarian follicle during ovulation, driving the apoptosis of non-dominant follicles [12]. During ovulation, the surge in luteinizing hormone (LH) and the neovascularization within the follicle lead to ROS production by vascular endothelial cells and macrophages, providing the stimulation required for oocyte maturation and follicle rupture [13]. Based on the findings of Prasad S et al., it is essential to maintain physiological levels of reactive oxygen species (ROS) within each oocyte not exceeding 60 ng, in order to sustain the diplotene arrest of follicular cells. This optimal ROS threshold ensures appropriate regulation of follicular development and exerts pivotal effects during oocyte maturation and embryo development, thereby preserving the developmental competency of oocytes [14].

Under normal circumstances, follicular cells and FF contain antioxidant enzymes and molecules that maintain a dynamic equilibrium with the oxidative system, keeping cellular ROS levels within physiological ranges, to ensure the proper functioning of oocytes and granulosa cells. However, when ROS and antioxidant systems become imbalanced in FF, ROS may exert detrimental effects on oocytes in the form of oxidative stress, identified as a significant factor that may adversely affect the outcomes of ART. During in vitro culture, oocytes are exposed to a high-oxygen environment and lack the complex protective mechanisms provided by free radical scavengers and antioxidant enzymes, likely leading to an imbalance between ROS and antioxidants, resulting in reduced in vitro culture success rates [15]. Research has shown that, in populations undergoing in vitro fertilization and embryo transfer (IVF-ET), there is a significant increase in the concentration of 8-hydroxy-2′-deoxyguanosine (8-OHdG), a DNA oxidative product, in FF from follicles with high rates of degenerate oocytes (poor oocyte quality). Furthermore, oocytes retrieved from follicles with elevated 8-OHdG concentrations exhibited lower fertilization rates [16]. High levels of ROS in FF can lead to damage to DNA, cellular scaffolding, and cell membranes, resulting in decreased oocyte quality and infertility [17,18]. In the context of IVF, excessive ROS in FF has also been associated with poorer pregnancy outcomes [19,20].

## 3. Oxidative Stress Biomarkers in Follicular Fluid

The body’s antioxidant system can be divided into two categories: enzymatic antioxidant systems, including superoxide dismutase (SOD), glutathione peroxidase (GSH-Px), and catalase (CAT); and non-enzymatic antioxidant systems, such as glutathione, melatonin, vitamin C, and vitamin E. Total antioxidant capacity (TAC) is also frequently used to represent the collective antioxidant capacity of enzymes and non-enzymatic antioxidants. When there is an imbalance between oxidation and the antioxidant system, reactive oxygen species (ROS) can directly or indirectly oxidatively damage DNA, proteins, and lipids, leading to gene mutations, protein denaturation, and lipid peroxidation, and ultimately affecting normal cellular functions. Therefore, markers such as malondialdehyde (MDA), lipid peroxidation (LPO), advanced oxidation protein products (AOPP), and 8-OHdG are commonly used to assess oxidative damage. Michalina et al. compiled studies from 2012 to 2022 in the field of female reproduction and identified the most commonly used oxidative stress markers in female reproduction as ROS, 8-OHdG, MDA, TAC, and glutathione (GSH) [21]. Research by Iman et al. suggested that oxidative stress biomarkers such as 8-OHdG, TAC, and MDA are associated with reproductive hormones and ART pregnancy outcomes, potentially serving as valuable tools for evaluating clinical characteristics in IVF patients [22].

Direct measurement of ROS levels in follicular fluid, a microenvironment crucial for oocyte development, holds significant importance. It provides key information about the health status of oocytes and granulosa cells, offering valuable predictive insights for physicians and patients before oocyte fertilization. Analyzing ROS levels in FF can be used to assess a patient’s fertility potential, aiding in the selection of appropriate ART strategies to enhance success rates. Furthermore, ROS analysis can help identify potential factors contributing to infertility, such as declining oocyte quality or damaged granulosa cells, guiding physicians toward implementing more targeted treatment measures. However, this method of assessment comes with challenges and issues. First, the timing of sampling is critical, as the ROS levels in FF may vary at different stages of follicular development. Second, the accuracy and reliability of the technique must be ensured to avoid misdiagnosis or misguidance of patients. Most importantly, ROS testing results often need to be combined with other clinical and biochemical factors to comprehensively evaluate a patient’s fertility prospects, as successful ART depends on a multitude of factors beyond oocyte and granulosa cell status. Therefore, direct measurement of ROS in FF holds great potential in the field of assisted reproduction, offering more personalized and accurate treatment plans for infertility patients. However, further research and exploration are required to fully harness its potential for improving fertility success rates.

### 3.1. Total Antioxidant Capacity (TAC)

TAC refers to the overall level of antioxidants and antioxidant enzymes in the body, such as vitamin C, vitamin E, carotenoids, superoxide dismutase (SOD), glutathione peroxidase (GSH-PX), and catalase (CAT), which protect cells and organisms from the oxidative stress caused by reactive oxygen species. Therefore, the measurement of total antioxidant capacity (TAC) in cells or body fluids has significant biological significance.

The impact of follicular fluid TAC on oocyte quality and assisted reproductive outcomes remains controversial. Oyawoye et al. found that TAC levels were not significantly associated with the presence of oocytes in the follicles, but higher TAC levels were observed in the follicular fluid of successfully fertilized oocytes [23]. On the other hand, Appasamy et al. only detected follicular fluid TAC and found no correlation with ovarian response or pregnancy outcomes [24]. Pasqualotto et al. tested follicular fluid TAC in 41 IVF cycles and found no correlation between TAC levels and patient age, oocyte maturity, fertilization rate, or embryo quality. However, the TAC levels in the follicular fluid of pregnant patients were significantly higher than those of non-pregnant patients [25]. The contradictory results may be attributed to the small sample size, individual variations among the patients, and the different methods used for TAC measurement. In summary, there is currently no evidence that TAC can be used as a predictive indicator of fertilization rate and pregnancy outcomes.

### 3.2. Malondialdehyde (MDA)

MDA is a reactive carbonyl substance generated in the body when oxygen free radicals attack polyunsaturated fatty acids in cell membranes, thereby initiating lipid peroxidation. MDA is highly cytotoxic and can cause cross-linking polymerization of proteins and other macromolecules, leading to structural and functional changes in cell membranes. Therefore, MDA can reflect the degree of cellular membrane lipid peroxidation and is commonly used as an indicator for measuring the level of oxidative stress.

Debbarh et al. found that the MDA levels in follicular fluid significantly increased from 37 years of age, and compared with younger patients under the age of 37, the high-age group (≥37 years) had significantly higher levels of MDA in follicular fluid. The researchers also measured indicators such as SOD, GSH, and catalase in the follicular fluid and believed that, with the gradual onset of reproductive aging from 37 years of age, the efficiency of reactive oxygen species clearance in follicular fluid decreased, with the antioxidant pattern changing accordingly [26].

Studies have found a negative correlation between MDA levels in follicular fluid and blastocyst formation rate, suggesting that elevated MDA and decreased antioxidant capacity in follicular fluid can affect fertilization rates and subsequent embryonic development [8]. MDA has also been found to be negatively correlated with the rate of high-quality embryos as a marker of oxidative stress [27]. However, Celik et al. concluded that the level of MDA in the follicular fluid was not related to fertilization outcome and pregnancy results [26]. In addition, in patients with PCOS, MDA in follicular fluid was a key factor affecting embryo quality, making MDA a predictive factor for embryo quality in PCOS patients [25].

Clearly, the studies on the relationship between malondialdehyde (MDA) in follicular fluid and oocyte quality and assisted reproduction outcomes are inconsistent, and this discrepancy is likely attributable to variations in sample size and study populations. As a crucial marker of membrane lipid oxidation, significantly increased levels of MDA in follicular fluid reflect an imbalance in oxidative and antioxidative systems, potentially impairing oocyte developmental competence.

### 3.3. 8-Oxodeoxyguanosine (8-OHdG)

8-Oxodeoxyguanosine (8-OHdG) is an oxidative derivative of deoxyguanosine and is the most common base modification in DNA damage induced by exposure to radiation, hydroxyl radicals, superoxide anions, or peroxynitrite [26]. During the process of ROS-mediated DNA oxidation, guanosine, being the most vulnerable base in DNA nucleotides, is directly attacked by hydroxyl radicals and superoxide anions, resulting in the formation of the oxidative adduct 8-OHdG. Consequently, 8-OHdG serves as a biomarker for oxidative stress (OS).

Studies have shown that in infertile women undergoing in vitro fertilization (IVF), there is a negative correlation between the concentration of 8-OHdG in granulosa cells and fertilization rates, as well as embryo quality [28]. Tamura et al. determined the percentage of degenerated oocytes and 8-OHdG levels in follicular fluid from 54 patients who underwent IVF-ET. The results showed that the 8-OHdG levels in the follicles of women with a high rate of oocyte degeneration (≥30%) were significantly higher than those in women with a low rate of degeneration of oocytes (<30%). The results demonstrated a positive correlation between 8-OHdG levels in FF of women undergoing IVF and the rate of oocyte degeneration [16]. Additionally, patients with endometriosis, a condition associated with oxidative stress, exhibited higher levels of 8-OHdG in their follicular fluid [4]. Research by Sándor et al. has shown that, in patients undergoing IVF, there is a negative correlation between 8-OHdG levels in granulosa cells and follicular fluid and oocyte maturation rates, along with further negative correlations between 8-OHdG levels in follicular fluid and the numbers of fertilized oocytes and embryos [29]. These findings collectively suggest that 8-OHdG levels, particularly in follicular fluid, can serve as a crucial indicator for predicting oocyte quality and assisted reproductive outcomes.

### 3.4. Advanced Oxidation Protein Products (AOPP)

Advanced oxidation protein products (AOPP) are oxidation-modified products of plasma proteins formed as a result of attack by reactive oxygen species (ROS). AOPPs serve as a reliable indicator reflecting protein damage due to oxidative stress within the body. Elevated levels of AOPPs in follicular fluid indicate an increase in non-functional proteins and enzymes, potentially affecting normal metabolic processes within FF and adversely impacting oocyte development and maturation. Therefore, studying the relationship between AOPPs in follicular fluid and oocyte quality, as well as the outcomes of assisted reproductive pregnancies, is meaningful.

Research has shown that AOPP levels in follicular fluid increase with age in women, possibly due to elevated ROS levels and increased granulosa cell apoptosis in older individuals. Elevated AOPP levels in follicular fluid are significantly negatively correlated with oocyte maturation rates, fertilization rates, cleavage rates, and high-quality embryo formation. Non-pregnant women exhibit significantly higher AOPP levels in follicular fluid compared to pregnant women. These findings suggest that high levels of AOPP in follicular fluid may adversely affect oocyte and early embryo development, thereby influencing the outcomes of IVF-ET treatments [30].

Studies on AOPP in follicular fluid remain relatively limited. However, endometriosis, a disease closely associated with oxidative stress and inflammatory reactions, has been shown to significantly increase AOPP concentrations in peritoneal fluid and serum [31,32]. In patients with endometriosis, follicular fluid AOPP levels were found to be significantly higher than in control groups, with a significantly lower rate of blastocyst formation [33]. This indicates that AOPPs may serve as a potential marker for predicting oocyte quality and treatment outcomes in endometriosis patients.

### 3.5. Superoxide Dismutase (SOD)

Superoxide dismutase (SOD) is a class of metal enzymes, with the SOD family present in various tissues, including the ovaries. Its primary role is in the maturation of oocytes. SOD catalyzes the dismutation of superoxide anions (O^2−^) into hydrogen peroxide (H_2_O_2_) and oxygen (O_2_). H_2_O_2_ is then detoxified into water (H_2_O) and oxygen (O_2_) by catalase (CAT) or glutathione peroxidase (GPX), thus protecting the oocytes. The SOD family includes three different subtypes with distinct localization and functions: SOD1 (copper-zinc superoxide dismutase, CuZn-SOD), which is widely present in the cytoplasm of eukaryotic cells and neutralizes superoxide anions in oocyte cytoplasm; SOD2 (manganese superoxide dismutase, MnSOD), located in mitochondria and particularly important for removing ROS produced by cytokines and inflammation in mitochondria; and SOD3 (extracellular superoxide dismutase, EC-SOD), a secretory protein primarily responsible for clearing extracellular superoxide radicals. EC-SOD is the only subtype found in the zona pellucida [34].

SOD has been detected in follicular fluid in various mammalian species, such as humans, cows, and mice. In cows, all three subtypes of SOD have been identified in follicular fluid, with total SOD activity showing a negative correlation with follicle size [35]. Studies have demonstrated that Sod1-null mice exhibit reduced fertility, while Sod2-deficient mice die within three weeks of birth. However, when ovaries from Sod2-deficient mice are transplanted into wild-type mice, they can produce offspring. This confirms the importance of SOD1 in female reproduction, although SOD2 is not essential for ovarian function [36]. In older women, total SOD activity in follicular fluid decreases and is positively correlated with normal fertilization rates and high-quality embryo formation. This suggests that SOD may have an influence on oocyte quality [27]. These studies suggest that as ovarian aging progresses, leading to follicle depletion and decreased oocyte quality, follicular ROS levels gradually increase. This increase may be associated with an elevation in ROS production or a decrease in the activity of antioxidants, particularly SOD and catalase, which serve as the primary line of defense against oxidative stress.

### 3.6. Glutathione (GSH)

Glutathione (GSH) is a molecule composed of glutamic acid, cysteine, and glycine and is widely distributed in the body. GSH exists in two forms within the body: reduced (GSH) and oxidized (GSSG). The reduced form, GSH, is the predominant active state. GSH molecules feature an active thiol group (-SH), which can activate various enzymes and participate in several critical biochemical reactions within the body. GSH can also bind to certain drugs (such as acetaminophen), toxins (such as free radicals, mustard gas, and heavy metals like lead, mercury, and arsenic), providing antioxidant and detoxification functions.

Studies have found that there is no significant correlation between the concentration of GSH in follicular fluid and fertilization rates, embryo quality, or pregnancy outcomes [37]. Some research has shown that total GSH levels in FF decrease in cases of low fertilization rates following intracytoplasmic sperm injection (ICSI). Additionally, GSH levels in the FF of patients with endometriosis were found to be lower [24]. This suggests that GSH levels in follicular fluid may still have some impact on the developmental potential of embryos. Furthermore, glutathione reductase can convert oxidized glutathione (GSSG) back into reduced glutathione (GSH). The level of glutathione reductase in follicular fluid may also contribute to maintaining or increasing GSH levels. Therefore, the relationship between GSH levels in follicular fluid and oocyte quality and assisted reproductive outcomes may not be straightforward.

When synthesizing the aforementioned information, we identified MDA and 8-OHdG as two relatively reliable indicators for assessing ROS levels in follicular fluid. Despite some research existing in this field, there are circumstances that may render the results inconclusive or even produce contradictory effects. First, a limitation in sample size could be one of the contributing factors to these situations. Due to a limited number of samples, research results may be subject to statistical sample bias, thereby leading to conclusions that lack representativeness. In order to more precisely assess the feasibility of these indicators, future studies may need to increase the sample size to enhance the reliability of the research. Second, the majority of studies analyzed pooled follicular fluid from multiple follicles of the same patient rather than individual follicular fluid. Considering the heterogeneity of follicles, this may also be one of the reasons for the lack of consistency in results. Research focusing on the follicular fluid of individual follicles may contribute to a deeper understanding of the variation in ROS levels. Shifting the research focus to individual follicles holds the potential to provide more specific and detailed insights, aiding in revealing the relationship between ROS levels and follicular health.

## 4. Measures to Improve Follicular Fluid Oxidative Stress Status

Excessive levels of ROS and oxidative stress in follicular fluid can potentially have adverse effects on oocyte development and quality, subsequently impacting pregnancy outcomes. In recent years, several small molecules with antioxidant properties have been found to mitigate oxidative stress damage. Here, we provide an overview of the use of antioxidants such as melatonin, coenzyme Q10, resveratrol, and NAC in the in vitro maturation of oocytes, oocyte cryopreservation, and assisted reproductive technology (ART) procedures for elderly and ovarian dysfunction patients, aiming to improve embryo development.

### 4.1. Melatonin

Melatonin, secreted by the pineal gland, is an amine hormone with potent neuroendocrine regulatory activity. It also acts as an endogenous free radical scavenger, possessing strong antioxidant capabilities. Due to its lipophilic and hydrophilic properties, melatonin easily crosses cell membranes to exert its effects.

Studies have shown that follicular fluid contains high levels of melatonin, with its concentration increasing with follicle growth, indicating a potential influence on mammalian ovarian and reproductive function [38]. As a potent free radical scavenger, melatonin ensures the proliferation of granulosa cells and the normal development and maturation of oocytes, effectively suppressing ovarian aging.

As presented in Table 1, the impact of the antioxidant melatonin on assisted reproductive outcomes in various species has been extensively studied. In various animals such as mice [39,40], pigs [41,42], and sheep [43,44], studies have shown that the addition of melatonin to culture media can improve oocyte maturation, fertilization rates, and blastocyst formation (blastocyst cell count). Additionally, in studies of pig oocyte maturation in vitro, the addition of melatonin was found to suppress the effects of substances inducing oxidative stress, such as bisphenol A (BPA) [45] and aflatoxin B1 (AFB1) [46]. Interestingly, Jia et al. found that adding melatonin to culture media not only reduced oxidative stress and improved pre-implantation embryo development but also enhanced fetal development within the uterus, as well as rescuing impaired glucose metabolism in IVF offspring [47]. In human oocytes in vitro maturation studies, melatonin was shown to enhance oocyte development, leading to the formation of more blastocysts, a significantly reduced rate of aneuploidy, and the successful birth of three healthy babies in subsequent clinical trials. Zou et al. cultured a total of 105 oocytes in IVM medium containing 10 μmol/L melatonin. Subsequently, ICSI was performed, and the lysed embryos and blastocysts were cultured, and finally 33 blastocysts were formed, including 19 high-quality blastocysts, which was significantly more than the one high-quality blastocysts in the control group. Therefore, these studies showed that melatonin contributes to the formation of high-quality blastocysts. Sequencing results indicated that melatonin primarily exerts its effects by improving mitochondrial function [48]. In a study collecting follicular fluid from women undergoing IVF-ET, a significant negative correlation was found between melatonin concentration and 8-OHdG levels [16]. Moreover, melatonin can reduce oxidative damage to mouse ovaries caused by exogenous chemical toxins such as chemotherapy drugs. Adding melatonin as a cryoprotectant can improve the effectiveness of human oocyte cryopreservation by inhibiting oxidative damage and enhancing oocyte membrane permeability [49]. Clinical administration of melatonin to patients can reduce the levels of 8-OHdG in oocytes of infertile women and improve IVF fertilization rates [16]. Research suggests that melatonin may improve the oxidative stress status of in vitro cultured oocytes and embryos during assisted reproduction processes through its antioxidant capabilities and the MT1/AMPK pathway, ultimately enhancing oocyte and embryo developmental competence and improving clinical treatment outcomes [50,51]. However, the exact mechanism of its action remains unclear. Concerns about potential adverse reactions from long-term administration of melatonin have arisen, due to its widespread effects on organism metabolism and physiological activities [52]. Additionally, a few studies have reported the ineffectiveness of melatonin. Fernando et al., in a study of 150 participants, found that increased melatonin concentrations in serum and FF did not significantly improve the number and quality of oocytes and embryos, or the clinical pregnancy rate [53].

### 4.2. Coenzyme Q10 (CoQ10)

Coenzyme Q10 (CoQ10) is a lipid-soluble quinone compound that participates in ATP synthesis in the mitochondrial respiratory chain. It functions as an antioxidant within the mitochondrial respiratory chain, inhibiting lipid peroxidation and DNA oxidation. One of the characteristics of human aging is the decline in CoQ10 concentration in tissues. Studies have shown that severe deficiency of CoQ10 (<20% of normal levels) in an organism may lead to significant bioenergetic insufficiency, but this is not necessarily accompanied by a noticeable antioxidant stress response. On the other hand, intermediate levels of CoQ10 deficiency (between 30% and 45% of normal levels) are commonly observed in age-related CoQ10 deficiency syndrome and may cause moderate bioenergetic insufficiency along with a significant increase in the production of reactive oxygen species (ROS), lipid oxidation, and cell death. The decrease in plasma CoQ10 levels in humans is associated with reduced gonadal function and changes in other steroid hormone levels [59]. It decreases with age and is accompanied by an increased rate of embryonic aneuploidy [60]. Supplementation of CoQ10 may improve mitochondrial function, enhance oocyte quality, and improve pregnancy outcomes in infertility patients. In animal experiments, treatment of 52-week-old mice with CoQ10 significantly increased the number of ovulations, reduced mitochondrial membrane potential and mitochondrial copy numbers, restored the citrate/ATP ratio, and increased the number of offspring [61]. Human studies have shown that oral supplementation of CoQ10 in infertile women undergoing assisted reproductive technology (ART) procedures can increase clinical pregnancy rates [62]. However, some studies suggested that supplementation with CoQ10 (600 mg/d for 2 months before ovarian stimulation) can improve fertility. Compared to 93 participants in the control group, 76 women treated with CoQ10 (600 mg/d for 2 months before ovarian stimulation) showed increased ovarian responsiveness, fertilization rate, and number of high-quality embryos in young women with diminished ovarian reserve, but no significant improvement was observed in clinical pregnancy rates, miscarriage rates, and live birth rates [63].

During the in vitro maturation (IVM) process, CoQ10 improves the outcome of IVM by enhancing mitochondrial function in immature oocytes. A study by Ma et al. [64] in 2020 demonstrated that supplementation of CoQ10 in older women significantly improved oocyte maturation during the IVM process (82.6% vs. 63.0%, *p* = 0.035) and significantly reduced the rate of post-meiotic aneuploidy (36.8% vs. 65.5%, *p* = 0.020). However, similar effects were not observed in younger women [64].

### 4.3. Resveratrol

Resveratrol is a non-flavonoid polyphenolic compound and a natural activator of silent information regulation (Sirtuin). It improves mitochondrial function and has antioxidant and anti-aging effects. Adding an appropriate concentration of resveratrol during the in vitro development of oocytes and embryos can clear excess ROS, increase GSH levels, reduce apoptosis, and regulate the expression of apoptosis-related genes. For oocytes matured in vitro, pretreatment with resveratrol helped reduce ROS levels in pig oocytes, improving the subsequent development after in vitro fertilization or parthenogenetic activation [65]. In a study of female mice at 48–52 weeks of age and 64 women aged 38–45 years who underwent intracytoplasmic sperm injection (ICSI), Liu et al. found that adding 1.0 μM resveratrol to the IVM culture medium for human and mouse oocytes improved oocyte maturation rates and oocyte quality, significantly reducing the proportion of abnormal oocytes [66]. Adding resveratrol to the culture medium for bovine embryos effectively reduced ROS levels, increased ATP production, and reduced lipid content in embryos, thereby improving their in vitro development [67]. Table 2 summarizes the extensive research on the impact of the antioxidant resveratrol on assisted reproductive outcomes in various species. Through a study of 61 patients with PCOS, Bahramrezaie found that oral administration of 40 mg/day resveratrol to PCOS patients undergoing ICSI increased the rate of high-quality oocytes and high-quality embryos. This may function through altering serum levels of sex hormones and the expression of VEGF and HIF1 genes in granulosa cell angiogenesis pathways [68]. However, the biological effects of resveratrol are dose-dependent, and both too low and too high concentrations may result in no effect or even adverse effects, especially at high concentrations, which may lead to conditions such as goiter.

Itami et al. found that resveratrol enhanced mitochondrial function by activating SIRT1, increasing ATP levels, and improving oocyte quality in pigs [69]. Similar mechanisms were confirmed in bovine oocytes by Takeo et al. [70]. For aging animals, adding resveratrol to the culture medium for oocytes from pigs [71], cows [65], and mice [66] increased the expression of SIRT1, enhanced mitochondrial function, and improved the quality of oocytes. Resveratrol can improve the function of granulosa cells by increasing the activity of SIRT1, enhancing their antioxidant capacity and anti-apoptotic ability.

Additionally, resveratrol contributes to the protection of vitrified-warmed embryos. Research has shown that 25 μM resveratrol can reduce oxidative stress in vitrified oocytes and alleviate abnormal mitochondrial distribution after vitrification, indicating that resveratrol can reduce freezing damage in mouse oocytes [72].

**Table 2:** Impact of the antioxidant resveratrol on assisted reproductive outcomes in various species.

Species	Protocol	Treatment Outcome
Mouse	Addition of 1.0 μM resveratrol in IVM culture medium	Improved oocyte maturation rates and oocyte quality, significantly reducing the proportion of abnormal oocytes [66].
	Addition of 25 μM resveratrol in vitrification medium (ES, VS), warming medium (TS, DS, and HM), and post-warming medium (IVF medium)	Reduced oxidative stress in vitrified oocytes and alleviated abnormal mitochondrial distribution after vitrification [72].
Pig	Addition of 2 μM resveratrol in IVM growth medium	Enhanced mitochondrial function by activating SIRT1, increasing ATP levels, and improving oocyte quality [69].
	Resveratrol at 2 μmol/L for 68 h in maturation medium	Significantly improved the quality of aged oocytes, including the assembly of meiotic apparatus, redistribution of cortical granules, and mitochondria in pig oocytes [71].
Cow	Addition of different concentrations of resveratrol (0, 0.1, 1.0, or 10.0 μM) in maturation medium	Reduce ROS levels, improving subsequent development after in vitro fertilization or parthenogenetic activation [65].
	Cultured in medium containing 0 or 0.5 µM resveratrol for 1 or 5 days	Enhanced mitochondrial functions via SIRT1 expression, reduced lipid content via beta-oxidation, improved the rate of embryonic development to the blastocyst stage, and improved blastocyst cryotolerance [67].
	Cultured in TCM-199 medium supplemented with 10% FCS and 0 or 20 µM resveratrol	Improved the quality of oocytes by improving mitochondrial quantity and quality, enhanced SIRT1 protein expression in oocytes, and improved fertilization [70].

### 4.4. N-Acetylcysteine (NAC)

NAC, as a precursor of L-cysteine, which is required for glutathione biosynthesis, can increase intracellular glutathione levels [73]. It has the ability to scavenge free radicals and reduce oxidative stress damage. NAC can also directly react with free radicals such as OH^−^ and nitrogen dioxide (NO_2_), reducing ROS production [74,75].

Several studies have reported the application of NAC in in vitro maturation (IVM) culture systems for animal oocytes. Sun et al. [76] demonstrated that adding 1 mM NAC to the IVM medium for bovine oocytes for 8 h significantly increased oocyte maturation rates and blastocyst formation rates. This process was achieved through preventing apoptosis and reducing ROS during maturation [76]. Whitaker et al. found that adding NAC to an in vitro maturation system for pig oocytes reduced DNA fragmentation, increased parthenogenetic activation, and subsequently improved blastocyst formation rates [77,78]. Wang et al. demonstrated that adding NAC to the in vitro maturation medium for mouse oocytes reduced spindle defects, reduced intracellular H_2_O_2_ concentrations, lowered ROS levels, increased ATP levels, prevented abnormal mitochondrial distribution, improved oocyte quality, and increased blastocyst formation rates [79,80]. Additionally, NAC improved oxidative stress in vitrified-warmed oocytes from mice, enhancing their developmental capacity [81].

The effects of NAC have also been validated in clinical trials. Li et al. found that oral NAC supplementation improved blastocyst quality in older women undergoing IVF/ICSI cycles [82]. A systematic review and meta-analysis of randomized controlled clinical trials showed that, in patients with PCOS, oral NAC significantly increased ovulation rates, pregnancy rates, and live birth rates [83].

However, the response to NAC treatment varies significantly among oocytes from follicles of different diameters in pigs (*p* < 0.05). Overall, NAC promotes in vitro maturation of oocytes from small antral follicles and exhibits a clear dose-dependent effect, promoting granulosa cell expansion and first polar body extrusion, while significantly increasing total GSH content and the expression of antioxidant genes SOD and CAT in oocytes from small antral follicles. However, NAC treatment has no significant effect on ROS levels in oocytes from large or medium antral follicles [84].

Antioxidants, including vitamin C, have been employed in prior clinical trials. These trials typically utilized observational parameters such as oocyte maturation rate, fertilization rate, rate of high-quality embryos, clinical pregnancy rate, and implantation rate. However, there is a paucity of reports regarding the ultimate outcome, namely the live birth rate, and the sample sizes in these studies were relatively limited.

To gain insights into potentially enhancing treatment outcomes, particularly for patients with ovarian dysfunction or advanced age in clinical assisted reproduction, Table 3 presents the effects of antioxidants in clinical trials on human oocyte and embryo development competence.

## 5. Conclusions and Prospects

The interplay of the oxidative/antioxidative system and metabolic products within FF significantly influences follicular development, oocyte maturation, and subsequently the outcomes of ART. The acquisition of follicular fluid is a straightforward and non-invasive approach, rendering it an ideal means for evaluating the developmental potential of oocytes through the assessment of oxidative stress levels within the follicular milieu. Oxidative stress markers within FF, such as TAC, MDA, and 8-OHdG, among others, have pivotal significance in predicting the outcomes of ART. In the pursuit of measures and strategies aimed at ameliorating the follicular microenvironment or mitigating oxidative stress within oocytes, the utilization of antioxidants such as melatonin and coenzyme Q10 exhibits promise, particularly for women with diminished fertility. This offers valuable insights into potentially enhancing treatment outcomes, especially for patients afflicted with ovarian dysfunction or advanced age in the realm of clinical assisted reproduction.

In addition to the well-explored antioxidants in assisted reproduction research, NR, humanin, HNG, and others have demonstrated potential applications. The concentration of humanin in follicular fluid is positively correlated with ovarian reserve and clinical pregnancy rates [92], with downregulated humanin expression observed in the ovaries of PCOS patients. Further mechanistic studies confirmed that exogenous humanin supplementation significantly alleviated ovarian morphological abnormalities and systemic oxidative stress in a PCOS rat model [93], though human studies in this context are currently lacking. Additionally, the humanin derivative S14G-HN (HNG) exhibits a bioactivity 1000 times that of humanin [94], and mouse studies indicate that HNG can inhibit the oxidative stress, apoptosis, and ovarian damage induced by D-gal, promoting ovarian autophagy [95]. However, its role in human assisted reproduction has not been studied. Moreover, other bioactive molecules have shown potential applications in assisted reproduction. The antioxidant NR has been reported to partially rescue the irreversible arrest phenotype occurring between the 3-8 cell stages in IVF, showcasing significant potential [96]. As a precursor to NAD+, NMN has demonstrated the potential to improve oocyte quality by restoring intracellular NAD+ levels. Recent studies suggest that NMN supplementation can enhance the quality of oocytes in aged animals, including increased ovulation rates, maintenance of normal spindle/chromosome structure, and promotion of meiosis and fertilization capacity [97,98,99,100]. However, direct implications for human assisted reproduction are still limited.

Nonetheless, the administration of antioxidants is not devoid of side effects, including adverse reactions closely linked to the female reproductive system. For instance, the prolonged usage of pharmacological doses of vitamin C and E has the potential to exert adverse effects on both ovarian and uterine functionality [101]. Indeed, certain antioxidants, such as vitamins A and E, have been identified in clinical trials as associated with heightened mortality rates [93]. This phenomenon may be explicable by the nuanced role of oxidative stress, which may possess latent beneficial functions under specific circumstances [52]. Conversely, the application of antioxidants is further confounded by the inconsistency in results and the overall suboptimal quality of clinical trials [102]. Hence, the necessity for additional randomized trials conducted in more extensive cohorts is underscored.

## Figures and Tables

**Table 1:** Impact of the antioxidant melatonin on assisted reproductive outcomes in various species.

Species	Protocol	Treatment Outcome
Mouse	Addition of melatonin at a concentration of 10^−6^ M in IVM medium	Enhanced cumulus–oocyte complex expansion, increased oocyte maturation rates, and decreased oocyte histone acetylation levels [54].
	Microinjection of 10^−7^ M melatonin	Markedly improved blastocyst rates, increased blastocyst cell numbers, reduced blastocyst apoptosis rates, and enhanced embryo implantation efficiency and neonatal birth rates [40].
	Melatonin at 10 μM for 6 h in culture medium	Increased HAS2 and PGR expression, indicating cumulus expansion and increased fertilization rates in IVF [39].
	Addition of 10^−6^ M melatonin in IVM medium	Significant improvement in mitochondrial function and blastocyst formation rates. Rescued fetal growth restriction and glucose intolerance, and enhanced energy expenditure in IVF mice [47].
Pig	Addition of melatonin at 4.3 × 10^−8^ M in IVM medium	Marked increase in oocyte maturation rates, and a significantly higher proportion of parthenogenetic activation of oocytes developing into blastocysts [41].
	Addition of melatonin at 10^−9^ M in maturation, fertilization, and embryo culture media	While melatonin added to maturation and fertilization media did not increase fertilization rates and blastocyst formation rates of porcine in vitro-fertilized oocytes, its addition to embryo culture medium increased glutathione (GSH) levels, accelerated embryo development, and improved the outcomes and quality of in vitro-produced (IVP) embryos [55].
	Addition of melatonin at 10^−9^ M in IVM medium	Significant increases in parthenogenetic blastocyst formation rates and total blastocyst cell numbers, increased cumulus expansion, possibly mediated by MT2 receptors [42].
Sheep	Addition of melatonin at 10^−7^ M in culture medium	Increased cumulus–oocyte complex expansion, higher cleavage rates, and blastocyst rates in parthenogenetically activated embryos, likely mediated by MT1 receptors [44].
Cow	Addition of melatonin at 10^−9^ M in IVM medium	Enhanced blastocyst formation rates and embryo quality, with reduced apoptotic cell counts [56].
	Addition of melatonin at 10^−9^ to 10^−7^ M in GV-stage oocyte IVM medium	Increased oocyte maturation rates, improved embryo development, and significantly increased average cell numbers produced after in vitro fertilization. The involvement of melatonin receptors in mediating this phenomenon has been demonstrated [57].
	Addition of melatonin at 10^−7^, 10^−9^, and 10^−11^ M in IVM medium	Enhanced oocyte fertilization and development capabilities, possibly regulated through improvements in organelle distribution, increased GSH and ATP levels, and enhanced expression of antioxidant genes, thereby promoting cytoplasmic maturation [58].

**Table 3:** Effects of antioxidants in clinical trials on human oocyte and embryo development competence.

Antioxidant	Protocol	Experimental Design	Experimental Results
Melatonin	Oral melatonin (2 mg/day) administered for at least 3 weeks before hCG trigger dose	In the no supplementation group, 78 were inseminated by ICSI and 19 by conventional IVF (cIVF). In the melatonin supplementation group, 83 cycles were inseminated by ICSI, and 14 by cIVF.	Increased fertilization rate and blastocyst quality after melatonin treatment [85].
	Daily oral administration of 3 mg or 6 mg melatonin from the start of controlled ovarian stimulation to the day of follicle puncture	40 patients were divided into 4 groups: Group 1 (control *n* = 10), group 2 (*n* = 10), women who did not take melatonin; group 3 (*n* = 10), women who took a daily dose of 3 mg of melatonin; and group 4 (*n* = 10), women who took a daily dose of 6 mg of melatonin.	Both melatonin doses improved oxidative balance in infertile patients’ follicles and oocyte quality, resulting in increased pregnancy/live birth rates [86].
	115 patients with low fertilization rates in the previous IVF-ET cycle treated with melatonin (600 mg/day)	115 patients were divided into two groups: 56 patients with melatonin treatment (3 mg/day) and 59 patients without melatonin treatment.	Significantly increased fertilization rates, with no statistical difference in pregnancy rates compared to the control group [16].
	150IVF/ICSI patients randomized into placebo, 2 mg/day melatonin, 4 mg/day melatonin, and 8 mg/day melatonin groups	150 were randomized to receive placebo (*n* = 36), melatonin 2 mg (*n* = 38), melatonin 4 mg (*n* = 36), or melatonin 8 mg (*n* = 40)	Although melatonin concentrations in follicular fluid significantly increased in the melatonin treatment group, there were no differences in oocyte maturation rates, fertilization rates, embryo quality, or clinical pregnancy rates [53].
	193 immature oocytes collected from hyperstimulated ovarian cycles, randomly divided into a 10 μmol/L melatonin-treated group and control group	193 immature oocytes were divided into two groups: 10 μmol/L MT (*n* = 105, M group) and no MT (*n* = 88, NM group)	Higher blastocyst formation rate, significantly reduced aneuploidy rate, and successful delivery of three healthy babies in the melatonin group [48].
Coenzyme Q10	Randomized pre-treatment with CoQ10 (200 mg, three times daily for 60 days) or no pre-treatment for 10 days before IVF-ICSI cycles in PCOS patients	76 participants treated with CoQ10 and 93 control participants without any additional treatment.	Coenzyme Q10 pre-treatment group had increased E2 peak levels, significantly higher retrieval of oocytes, fertilization rates, and high-quality embryos, leading to higher clinical pregnancy and live birth rates, although without statistical differences [63].
	Addition of 50 mmol/L CoQ10 to IVM culture medium for 24 h	166 immature human oocytes were obtained (GV stage) from 63 women (45 patients aged ≥ 38 years and 18 patients aged ≤ 30 years)	In older women, CoQ10 treatment significantly improved oocyte maturation rates and reduced oocyte and chromosomal aneuploidy rates. In younger women, there were no significant differences in these parameters [64].
Resveratrol	Immature oocytes from ICSI patients treated with 1.0 μm resveratrol for 24 and 36 h	A total of 75 GV oocytes from 64 patients >38 years of age were randomly divided into two groups: 1.0 μm resveratrol and DMSO supplemented IVM media group.	Improved oocyte maturation and oocyte quality, with a significant reduction in abnormal oocyte proportions [66].
	800 PCOS patients undergoing ICSI randomized into treatment with daily oral resveratrol (40 mg) from the start of the menstrual cycle until oocyte retrieval	62 patients were randomly assigned to two groups: took resveratrol 800 mg/day, or placebo for 40 days	Higher rates of high-quality oocytes and high-quality embryos. Possible reductions in serum total testosterone and LH levels, and increased TSH and FSH levels [68].
	Comparison of transplant outcomes between resveratrol-treated group (200 mg/day) and control group in IVF-ET patients	The RES group (204 cycles, 102 women) receiving resveratrol supplementation (200 mg/day) continuously was compared with the control group (7073 cycles, 2958 women)	Reduced clinical pregnancy rates and increased miscarriage risk in the resveratrol treatment group [87].
Vitamin C	Daily intake of vitamin C (500 mg/day) in women undergoing ART procedures	76 women (38 of them smokers, 38 non-smokers) were studied. Half the women (19 smokers and 19 non-smokers) were administered vitamin C. The control group consisted of the same number of smokers and non-smokers.	Significantly higher pregnancy rates in women who consumed vitamin C compared to the control group [88].
Vitamin E and Vitamin D3	Randomized allocation of infertile women planning ICSI into vitamin treatment group (vitamin E, 400 mg/day, vitamin D3, 50,000 IU/one in two weeks) and placebo group	105 PCOS infertile women scheduled for ICSI were enrolled to treatment group(*n* = 52) or placebo group (*n* = 53) for 8 weeks.	Increased implantation and clinical pregnancy rates in the vitamin treatment group [89].
Melatonin + Inositol + Folate	Starting from the day of GnRH administration, one group received 3 g melatonin + 4 g inositol + 200 mg folate, while the other received inositol with folate	65 women undergoing IVF cycles were randomized into two groups to receive myo-inositol plus folic acid plus melatonin (32 women, group A), and myo-inositol plus folic acid (33 women, group B)	The melatonin combination treatment group showed significantly increased oocyte maturation rates, higher clinical pregnancy rates, and implantation rates, though without statistical significance [90].
	Three months of treatment with 3 mg melatonin + 4 g inositol + 4 g folate in women who failed to conceive in IVF cycles due to poor oocyte quality	All 46 women were treated with myo-inositol and melatonin for 3 months. Results were compared to the previous IVF cycle.	Significant increases in oocyte maturation rates, fertilization rates, total number of transferred embryos, and embryo quality [91].
